# Light-induced cell damage in live-cell super-resolution microscopy

**DOI:** 10.1038/srep15348

**Published:** 2015-10-20

**Authors:** Sina Wäldchen, Julian Lehmann, Teresa Klein, Sebastian van de Linde, Markus Sauer

**Affiliations:** 1 Department of Biotechnology and Biophysics, Biocenter, University of Würzburg, Am Hubland, 97074 Würzburg, Germany

## Abstract

Super-resolution microscopy can unravel previously hidden details of cellular structures but requires high irradiation intensities to use the limited photon budget efficiently. Such high photon densities are likely to induce cellular damage in live-cell experiments. We applied single-molecule localization microscopy conditions and tested the influence of irradiation intensity, illumination-mode, wavelength, light-dose, temperature and fluorescence labeling on the survival probability of different cell lines 20–24 hours after irradiation. In addition, we measured the microtubule growth speed after irradiation. The photo-sensitivity is dramatically increased at lower irradiation wavelength. We observed fixation, plasma membrane permeabilization and cytoskeleton destruction upon irradiation with shorter wavelengths. While cells stand light intensities of ~1 kW cm^−2^ at 640 nm for several minutes, the maximum dose at 405 nm is only ~50 J cm^−2^, emphasizing red fluorophores for live-cell localization microscopy. We also present strategies to minimize phototoxic factors and maximize the cells ability to cope with higher irradiation intensities.

Fluorescence microscopy is the method of choice for the relatively non-invasive visualization of biomolecules in living cells because it allows selective and specific detection of molecules with high signal-to-background ratio. However, with increasing spatiotemporal resolution the prevention of photodamage effects in live-cell fluorescence microscopy becomes increasingly challenging. This is especially true for single-molecule sensitive fluorescence imaging and tracking experiments where photobleaching of the fluorophores sets the ultimate experimental limit. To use the limited photon budget efficiently in live-cell experiments and reduce photobleaching and phototoxicity, low irradiation intensities confined to micron-thin planes^1^, e.g., light-sheet and Bessel beam plane illumination microscopy, have been used also in combination with super-resolution structured illumination microscopy^2^,^3^,^4^.

Super-resolution microscopy by single-molecule detection and precise position determination (localization microscopy)^5^,^6^,^7^,^8^ achieves a higher spatial resolution but requires higher irradiation intensities in the kW cm^−2^ range, because switching and activation rates of fluorophores are mainly a function of the laser power applied^9^. Total-internal reflection fluorescence (TIRF) microscopy can be used to lower the penetration depth to merely the basal cell membrane. In order to image cell’s interior, on the other hand, epi- or highly inclined and laminated optical sheet (HILO)^10^ illumination are required. Nevertheless, independent of the excitation method used high irradiation intensities generate reactive oxygen species (ROS) through excited-state reactions of endogenous and exogenous chromophores that have a high potential to damage cellular components^11^.

If the cell cannot handle, i.e., repair, accumulating phototoxic events during irradiation, it will ultimately die. Unfortunately, so far live-cell localization microscopy largely ignored possible phototoxic effects^12^ or treated them only superficially likely due to the nonexistence of appropriate instrumentation for automated long term live-cell observation. Hitherto, in most studies it was investigated whether the cells are still adherent, changed their shape, or showed other apparent ill effects directly after super-resolution microscopy experiments^13^,^14^,^15^. Recently, it has been shown that yeast cells that appeared healthy directly after irradiation with a very low light-dose failed to divide when left overnight, whereas their non-imaged neighbors divided normally^16^. Even though the exact mechanism behind light-induced cell damage is still unclear and the irradiation sensitivity will undoubtedly vary among different cell types and irradiation wavelengths^17^,^18^,^19^, the reported results clearly demonstrate that the simple observation of the cell’s appearance directly after irradiation cannot be used as a meaningful photodamage marker.

A variety of non-radioactive cell proliferation assays can be used to estimate the number of viable eukaryotic cells^20^,^21^. The MTT assay^22^ is one of the most popular assays, which can be used to probe cellular metabolism. Here, the tetrazolium salt MTT (3-(4,5-dimethlythiazol-2-yl)-2,5-diphenyltetrazolium bromide) is reduced by cellular reducing equivalents, such as NADH and NADPH, to a blue formazan product^23^. The latter is used as indicator for cell viability and measurable via quantitative absorption spectroscopy, e.g., with a plate reading spectrophotometer^21^.

Here, we used an alternative approach to probe the cell viability after super-resolution microscopy experiments where typically single or only a few cells are irradiated with the required high intensities. We monitored cell survival of irradiated and non-irradiated cells for 20–24 hours and observed microtubule growth after wide-field illumination in epi- and HILO-mode with typical irradiation intensities (0–3 kW cm^−2^) and wavelengths (405–640 nm) used in PhotoActivated Localization Microscopy (PALM)^5^,^13^ and *direct* Stochastic Optical Reconstruction Microscopy (*d*STORM)^14^,^15^,^24^. We investigated the influence of irradiation intensity, wavelength, light-dose, fluorescence labeling, temperature, and reducing agent (ascorbic acid) on the viability of various labeled and unlabeled cultured cell lines.

## Results

### Degree of photodamage

**Figure 1:**
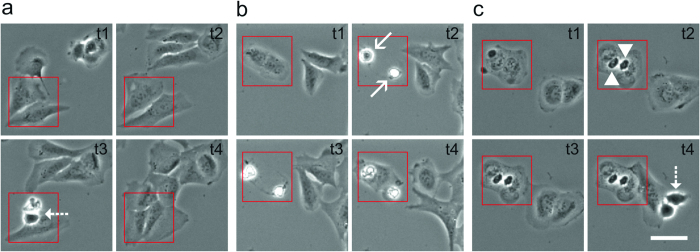
Classification of photodamage effects using U2OS cells in three categories. (**a**) Non-irradiated healthy cells (Supplementary Video 1), (**b**) apoptotic cells irradiated with an intensity of 0.49 kW cm^−2^ at 514 nm for 240 s (Supplementary Video 2), and (**c**) frozen cells irradiated with an intensity of 1.5 kW cm^−2^ at 514 nm for 240 s (Supplementary Video 3). Images were taken 1.15 h (t1), 6.15 h (t2), 10.30 h (t3), and 16.30 h (t4) after irradiation. The red rectangle in (**b**) and (**c**) shows the irradiated cells. Dashed arrows mark dividing cells, continuous arrows apoptotic cells, and arrowheads frozen cells. Scale bar, 50 μm.

We used U2OS, COS-7 and HeLa cells seeded in petri dishes with an imprinted 500 μm relocation grid, irradiated them under localization microscopy conditions (0–3 kW cm^−2^) and observed them afterwards for 20–24 hours under standard culture conditions using an automated cell observation system. As irradiation time we used 240 s, a typical acquisition time in localization microscopy, sufficient to record 12,000 frames with 20 ms integration time. We used high-power laser light sources to generate the required intensities over a large field of view (65.5 × 65.5 μm) in order to irradiate a few cells per experiment (Online Methods). The laser beams were confined with a rectangular field stop to ensure that only the cells present within the field of view are irradiated. Photodamage analysis was based simply on the fact whether the irradiated cells appear healthy and show cell division during the next 20–24 hour after irradiation or not. We classified the cells in three different photodamage categories. First, cells that appear healthy and show cell division after irradiation, termed ‘healthy’ (Fig. 1a and Supplementary Video 1), second, cells that show no or slowed cell division and detachment from the cover slip followed by cell death, termed ‘apoptotic’ (Fig. 1b and Supplementary Video 2), and third, cells that do not divide but appear completely immobile attached to the surface, termed ‘frozen’ (Fig. 1c and Supplementary Video 3). Frozen cells represent the most damaged type of cells through irradiation. For the sake of convenience we do not differentiate between apoptotic and frozen cells in the following. We simply count cells that survived irradiation and did further progress through the cell cycle like un-irradiated cells and cells that did not further divide and died.

### Effect of transfection, dye labeling, and cellular environment

**Figure 2:**
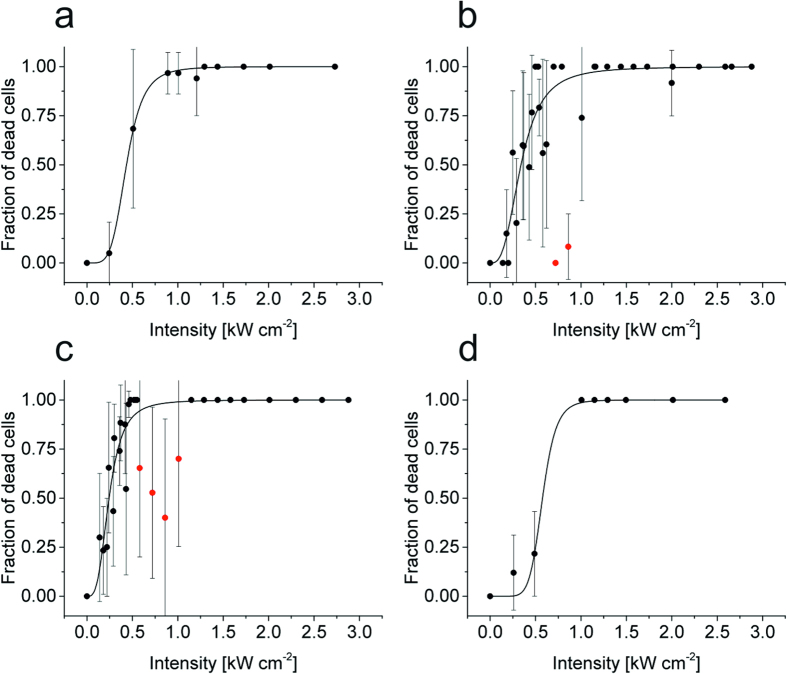
Dependence of cell survival on irradiation intensity at 514 nm for 240 s of differently modified U2OS cells. (**a**) Wildtype cells irradiated at 21 °C, (**b**) cells stably transfected with CLIP-H2B irradiated at 21 °C, (**c**) cells stably transfected with CLIP-H2B and labeled with TMR, irradiated at 21 °C and (**d**) cells stably transfected with CLIP-H2B irradiated at 37 °C. Red dots are masked data points and were not considered for fitting. Error bars are given as one standard deviation. For each data point 20–50 cells were irradiated (Table 1).

In each experiment we irradiated 20–50 cells and counted the number of ‘dead’ (apoptotic + frozen) cells (Table 1). First, we performed irradiation experiments with U2OS cells at different irradiation intensities (0–3 kW cm^−2^) at 514 nm for 240 s (Fig. 2 and Supplementary Fig. 2). We compared wildtype U2OS cells (Fig. 2a), U2OS cells stably transfected with CLIP-H2B (Fig. 2b), and U2OS cells stably transfected with CLIP-H2B and labeled with tetramethylrhodamine (TMR) (Fig. 2c), all irradiated at 21 °C. An additional experiment with U2OS cells stably transfected with CLIP-H2B was conducted at 37 °C (Fig. 2d). From the titration curves (Fig. 2) we determined the intensity where 50% of all irradiated cells died within 20–24 h after an irradiation time of 240 s (*I*_50_) and calculated the corresponding lethal dose LD50 (Table 1). These results indicate that transfection of cells increases the photodamage sensitivity already in the absence of fluorophores.

Labeling with fluorophores further promotes phototoxic effects. (Fig. 2c and Table 1). Transfection and labeling with an organic fluorophore (here TMR) that absorbs at the irradiation wavelength exhibit comparable photodamage efficiency using U2OS cells. Both, transfection and labeling lower the *I*_50_ irradiation intensity by 20–25% (Table 1) stepwise from 0.43 (untreated) via 0.34 (transfected) to 0.24 kW cm^−2^ (transfected and labeled). This implies that genetically modified fluorescently labeled cells require lower irradiation intensities to survive live-cell fluorescence imaging experiments. Furthermore, our finding demonstrates that besides ROS generation by fluorophore triplet states and additional toxicity potentially induced through the attachment of fluorophores to cellular molecules other important phototoxic sources exist.

In addition, our experiments show that cells have mechanisms to repair phototoxic effects when irradiated under ideal conditions at 37 °C and thus can stand higher irradiation intensities. Thereby, *I*_50_ increases by 35% to 0.58 kW cm^−2^. (Fig. 2d and Table 1). Furthermore, the photo-resistance of cells can be further improved by the addition of 100 μM ascorbic acid (AA) as a supplement to the imaging medium, thus increasing *I*_50_ by 26% to 0.54 kW cm^−2^ (21 °C) (Table 1 and Supplementary Fig. 1). After irradiation, the AA buffer was replaced by standard medium and the cells were observed in the live-cell recorder for the next 20–24 h.

### Wavelength and illumination-mode dependent phototoxicity

**Figure 3:**
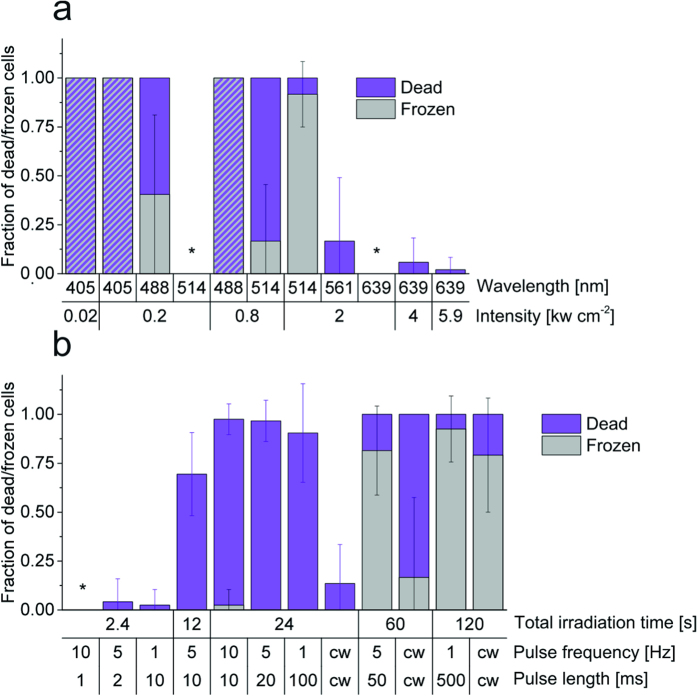
Wavelength, intensity, and irradiation-mode dependent phototoxicity. (**a**) Wavelength and intensity based phototoxicity upon cw irradiation for 240 s. (**b**) Cell death as a function of the irradiation light dose at 405 nm with an irradiation intensity of 0.02 kW cm^–2^ (pulsed vs. cw mode). Under pulsed irradiation conditions the total acquisition time was always 240 s. *No dead cells were observed. Error bars are given as one standard deviation. For each data point 10–50 cells were irradiated (Supplementary Table 1 and 2).

The energy of photons used to irradiate cells has a dramatic impact on phototoxicity (Fig. 3a and Supplementary Table 1). In particular, the photodamage efficiency increases with decreasing irradiation wavelength with the strongest phototoxic effect observed at 405 nm irradiation even at very low intensities, e.g., as typically used for photoactivation of fluorescent proteins in PALM experiments (0.02–0.05 kW cm^−2^)^13^. While at 0.2 kW cm^−2^ 488 nm irradiation for 240 s cells do not survive, 100% of cells survive when the wavelength is redshifted to 514 nm although the cells experienced the same light dose of ~48 kJ cm^−2^ (Fig. 3a and Supplementary Table 1). This small shift of 26 nm shows that cells exhibit a very distinct sensitivity related to the irradiation wavelength. But increasing the intensity at 514 nm to 2 kW cm^−2^ (~480 kJ cm^−2^) kills all cells, whereas 85% and 100% of all cells survive when irradiating with the same intensity and dose at 558 and 640 nm, respectively. Even when irradiating at 640 nm with intensities of 4–6 kW cm^−2^ (960–1,410 kJ cm^−2^) only weak effects on the cell survival rate are observed (<5% dead cells, Fig. 3a).

Next, we studied the effect of the irradiation dose at 405 nm. Irradiation with 0.02 kW cm^−2^ was applied at different pulse frequencies (continuous wave (cw), 10, 5, and 1 Hz) and pulse lengths (cw, 500, 100, 50, 20, 10, 2, and 1 ms) resulting in total irradiation times of 2.4–120 s (Fig. 3b and Supplementary Table 2). For pulsed irradiation the total acquisition time has been kept constant at 240 s. Our data show that pulsed and cw irradiation at 405 nm with 0.02 kW cm^−2^ corresponding to a total irradiation time of 60 seconds (1.2 kJ cm^−2^) is sufficient to kill virtually all irradiated U2OS cells (Fig. 3b). On the other hand, U2OS cells stand a 10^3^-times higher irradiation dose (1,410 kJ cm^−2^) at 640 nm undamaged (cw, 6 kW cm^−2^ for 240 s, Fig. 3a).

Interestingly, the strength of phototoxic effects for cw irradiation is lower than for pulsed excitation applying the same total irradiation time of 24 s, i.e., the same light dose. After cw irradiation with 0.02 kW cm^−2^ at 405 nm for 24 s (0.48 kJ cm^−2^) the survival fraction of cells is ~85%. In contrast, applying the same dose by pulsing the laser with 1 Hz pulse frequency and 100 ms pulse length, i.e., a recovery time of 900 ms between subsequent 100 ms irradiation pulses, decreases the survival fraction to ~10% (Fig. 3b). Increasing the pulse frequency and shortening the pulse length at constant total irradiation time of 24 s does not change the situation (Fig. 3b). This implies that U2OS cells rather withstand phototoxic stress through constant but short irradiation periods of 24 s (irradiation time/acquisition time: 24 s/24 s, 0.48 kJ cm^−2^) than repetitive irradiation pulses over a longer total acquisition time (24 s/240 s, 0.48 kJ cm^−2^) (Fig. 3b and Supplementary Table 2).

However, if cells are irradiated only for a total irradiation time of 2.4 s, short pulse lengths of 1 ms at a pulse frequency of 10 Hz allow total acquisition times of 240 s (corresponding to 48 J cm^−2^) and thus the observation of dynamic processes without any obvious phototoxic effect (Fig. 3b). Maintaining the light dose constant but increasing the pulse length only slightly, e.g., to 2 ms at 5 Hz pulse frequency, phototoxic effects are again detectable (Fig. 3b). These findings indicate that live-cell single-molecule localization microscopy experiments using 405 nm photoactivation can be performed for short total irradiation times using pulse frequencies of 10 Hz and pulse lengths of 1 ms or less at an irradiation intensity of 0.02 kW cm^−2^.

Irradiating cells for 60 or 120 s (1.2–2.4 kJ cm^−2^) leads to 100% dead cells, no matter whether cw or pulsed irradiation was applied. But pulsing increases the fraction of frozen cells, i.e., from 17% (60 s, cw) to 81% (60 s, 5 Hz) and 79% (120 s, cw) to 93% (120 s, 1 Hz). For the 24 s irradiation scenario, a small fraction of frozen cells (3%) was only observed when the pulse frequency was increased to 10 Hz (Fig. 3b and Supplementary Table 2). To summarize, our results demonstrate that the total irradiation time especially for 405 nm light has to be kept as short as possible in order to minimize phototoxic effects.

### Cell lines

**Figure 4:**
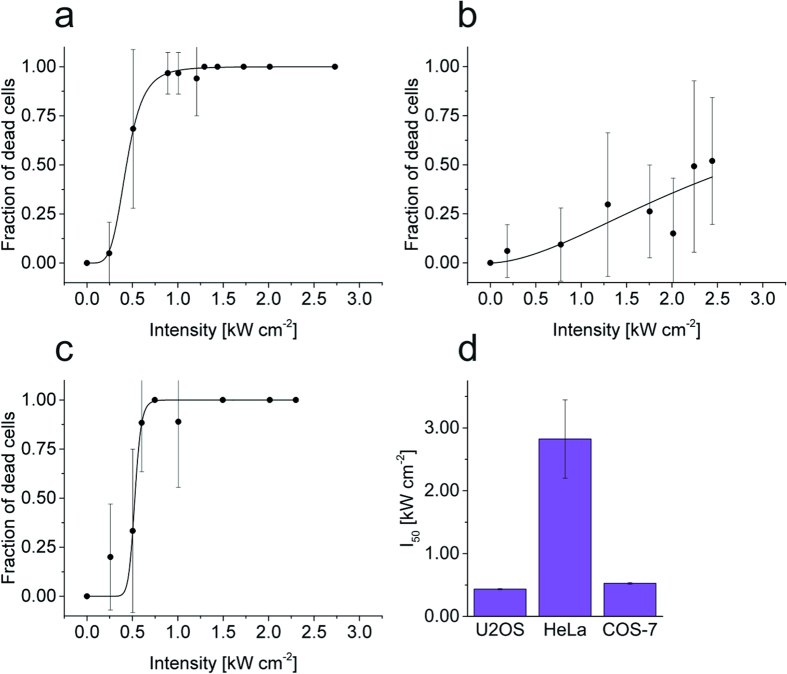
Cell line dependent photodamage efficiency. (**a**) U2OS, (**b**) HeLa, and (**c**) COS-7 cells were irradiated at 514 nm with varying intensity. (**d**) Corresponding *I*_50_ where 50% of the cells died (LD50). Error bars are given as one standard deviation. For each data point 20–50 cells were irradiated (Table 1).

Since it is expected that the photodamage sensitivity will vary between different cell lines, we performed additional experiments with COS-7 and HeLa cells (Fig. 4, Table 1). The results show that COS-7 cells exhibit a photo-sensitivity comparable to U2OS cells, whereas HeLa cells are substantially more resistant (Fig. 4d). The *I*_50_ value is about 0.5 kW cm^−2^ at 514 nm (120 kJ cm^−2^) for U2OS and COS-7 cells. The transition area between the lower and upper saturation levels—usually accompanied with huge variability in cell sensitivity (i.e., large standard deviations, compare Figs 2 and 4)—of U2OS and COS-7 are in the range of 0.25–0.75 kW cm^−2^. Here, the increased fluctuation in photo-sensitivity might be due to the fact that cells reside in different stages of the cell cycle. With the applied irradiation intensities at 514 nm, we were unable to achieve 100% dead HeLa cells (Fig. 4b). Even at the highest applied irradiation intensity of 2.5 kW cm^−2^ half of the irradiated HeLa cells survived. This demonstrates that HeLa cells exhibit the highest resistance to phototoxicity among all tested cell lines. The transition point *I*_50_ was determined to 2.8 kW cm^−2^ and is ~5–7 times higher than for COS-7 and U2OS.

### ‘Frozen’ cells

**Figure 5:**
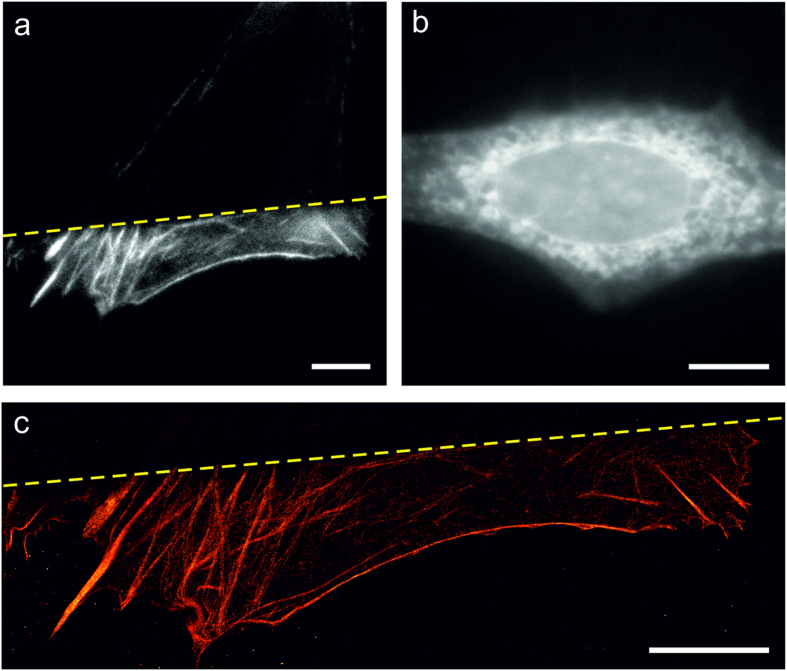
Imaging the cytoskeleton of a frozen U2OS cell without additional chemical fixation and permeabilization. (**a**) Epi-fluorescence image of f-actin. The upper part of a U2OS cell (yellow dashed line) was irradiated with 0.24 kW cm^−2^ at 405 nm for 240 s at 37 °C and stained with Alexa Fluor 647 phalloidin. Only the unirradiated lower part of the cell shows an intact actin network. F-actin in the upper part is completely destroyed. (**b**) Epi-fluorescence image of microtubules. The U2OS cell was irradiated with 0.05 kW cm^−2^ at 405 nm for 240 s at 37 °C and stained with SiR-Taxol^46^ (**c**) *d*STORM image of the actin structure shown in a). Scale bars, 10 μm.

So far, we hardly differentiated between ‘frozen’ and apoptotic cells (Fig. 1b,c). While in apoptotic cells too many phototoxic effects lead the cell to initiate cell death, frozen cells appear to die instantaneously without any residual mobility as if they would have been fixed by light (Fig. 1c). In fact, upon irradiation at 405 nm with an intensity of 0.24 kW cm^−2^ for 240 s (57.6 kJ cm^−2^) cells are not only fixed but additionally the membrane and cytoskeleton is destroyed (Fig. 5a).

Labeling of cells with Alexa Fluor 647 phalloidin followed by fluorescence imaging directly after irradiation without any additional fixation and permeabilization step demonstrates that only non-irradiated parts of the cell show typical actin filaments, irradiated parts show diffusive background fluorescence (Fig. 5a). Furthermore, the structure was super-resolved using *d*STORM (Fig. 5c). Here, it can be seen that at the edge of the illumination area actin filaments and bundles are sharply disrupted. Labeling of ß-tubulin in suchlike irradiated cells shows similar results, i.e., the membrane and microtubule network is depolymerized upon irradiation of cells at 405 nm with 0.05 kW cm^−2^ for 240 s at 37 °C (Fig. 5b).

### Microtubule dynamics as light sensor

**Figure 6:**
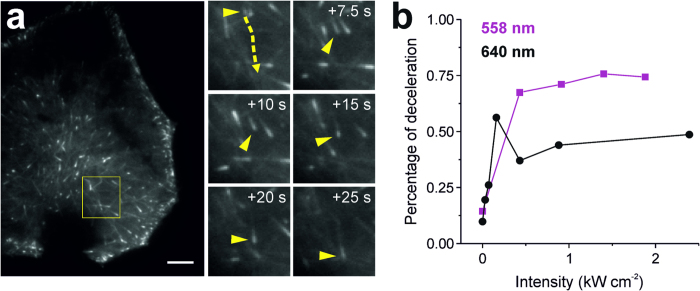
(**a**) Live-cell recording of EB1-N-YFP transfected HeLa cells. Movement of a single EB1-N-YFP molecule is shown. Dashed arrow in yellow indicates individual EB1 track, arrow heads depict the same EB1 molecule (Supplementary Fig. 5). (**b**) Deceleration of EB1 movement depends on the irradiation intensity at 558 nm (magenta) and 640 nm (black) (median).In absence of additional irradiation at 558 nm and 640 nm the microtubule growth speed decreases due to irradiation at the excitation wavelength of EB1-N-YFP at 488 nm with an intensity of <0.1 kW cm^−2^. Solid lines were used to guide the eye. Cells were irradiated at 37 °C for 225 s. The number of tracks analyzed per data point is given in Supplementary Table 3. Scale bar, 5 μm.

To identify already first signs of light-induced cell damage beyond the crude dead or alive criterion, we performed additional experiments tracking microtubule dynamics after irradiation. To this end, we used HeLa cells stably expressing YFP-tagged end binding protein 1 (EB1). EB1 localizes to microtubule plus ends and modulates their dynamics and interactions with intracellular organelles^25^,^26^,^27^. Hence, by tracking the movement of EB1-N-YFP the microtubule growth can be monitored (Fig. 6a). Here it has to be considered that the HeLa cells have been stably transfected and microtubule growth represents a central element of cell viability. Therefore, the irradiation resistance of the HeLa cells may be strongly affected (Fig. 2b and Table 1).

The speed of microtubule growth was measured with weak 488 nm irradiation, with an overall irradiation intensity <10 W cm^−2^ for 10 s (<100 J cm^−2^), before and after additional irradiation at 558 nm or 640 nm (Fig. 6b and Supplementary Fig. 5). The low irradiation dose allowed us to balance efficient read out of the YFP signal and phototoxic effects. All cells merely irradiated under these conditions survived (n = 15; Supplementary Table 3 and Supplementary Fig. 3). However, we observed a mean deceleration of microtubule growth from 59 to 53 μm min^−1^ upon irradiation solely at 488 nm i.e., a deceleration by 11%. Additional irradiation with 0.03 kW cm^−2^ at 640 nm for 225 s decelerates microtubule growth by 19% (Fig. 6b). Albeit strong phototoxic effects for irradiation at 640 nm are not expected (Fig. 3a), these results demonstrate that microtubule growth is sensitive to much weaker irradiation intensities. Upon further increase of irradiation intensity at 640 nm microtubule growth slows down by 40–50% of its initial speed (Fig. 6b). This value remains unaffected up to irradiation intensities of 10 kW cm^−2^ at 640 nm (Supplementary Table 3). For 558 nm irradiation microtubule growth speed decreases to a plateau with 70 to 76% of the initial speed applying irradiation intensities of up to ~2 kW cm^−2^ (Fig. 6b). After irradiation with intensities of ~5 kW cm^−2^ at 558 nm and higher, microtubule growth could not be measured anymore, because of the loss of microtubule primary structure and cell death, respectively (Supplementary Fig. 4).

## Discussion

In previous live-cell single-molecule localization microscopy studies the effect of irradiation and potential light-induced damage was judged after irradiation by the immediate appearance of the cells^13^,^14^,^15^. In this work, phototoxic effects were studied by observing cell viability after irradiation for a period of 20–24 h. Here, we discovered two different cell death mechanisms (Fig. 1). Apoptotic cells still showed motility directly after irradiation, but died some hours later, whereas frozen cells died instantaneously through irradiation. Therefore, it is hard to judge to which extent apoptotic cells were already damaged during imaging. However, it is certain that through imaging they were damaged to an extent that outreached the cells’ repair mechanisms, resulting in cell death later on.

Our results demonstrate that stable transfection of cells reduces photoresistance (Fig. 2 and Table 1), at which the degree depends on the transfected protein and its importance for cell viability. Thus, it has to be considered that genetically modified fluorescently labeled cells might require lower irradiation intensities to survive live-cell fluorescence imaging experiments. As expected, irradiation experiments with labeled cells indicate that ROS generated through fluorophore triplet states and subsequent singlet oxygen generation damage cells and reduce their photoresistance (Fig. 2 and Table 1)^28^,^29^. However, the degree of photodamage remains reasonable at least for standard organic fluorophores with low triplet quantum yields as used in our experiments. Thus, other processes such as the absorption of light by endogenous cellular chromophores and subsequent excited state reactions accompanied by generation of toxic substances, have to be taken into account to explain the observed phototoxic effects.

Generally, cells exhibit a very distinct irradiation sensitivity, i.e., the phototoxicity increases dramatically with decreasing irradiation wavelength (Fig. 3a)^30^. This is most impressively illustrated comparing the photodamage efficiency through irradiation at 488 nm and 514 nm with an intensity of 0.2 kW cm^−2^ (Fig. 3a). Here, a wavelength shift of only 26 nm suffices to dramatically change the survival rate. Fortunately, irradiation of fluorophores in the near infrared region at, e. g., 640 nm with an intensity <6 kW cm^−2^ without additional activation at 405 nm, can be considered innocuous^14^. However, after irradiation at 640 nm we observed a deceleration of microtubule growth speed (Fig. 6b) indicating that monitoring of microtubule growth speed is a very sensitive photodamage parameter.

Our data also clearly show that the fraction of apoptotic and frozen cells is particularly high at 405 nm irradiation even at a very low intensity of 0.02 kW cm^−2^ (Fig. 3a), the wavelength at which most photoactivatable fluorescent proteins have to be activated over time periods of several tens of minutes in PALM experiments^13^,^31^. Furthermore, we discovered that especially shorter wavelength photons can fix cells, permeabilize the plasma membrane and depolymerize the cytoskeleton (Fig. 5). If one would succeed to fix and permeabilize cells without any further cellular destruction by application of a short UV-light pulse one would have an elegant method at hand to freeze a specific cellular state during live-cell fluorescence imaging under low irradiation conditions.

One way to minimize phototoxic effects in PALM experiments is pulsed activation at 405 nm with short pulse lengths of 1 ms at 10 Hz pulse frequency applied for short total irradiation times of only a few seconds (Fig. 3b). In addition, TIRF microscopy can be used to confine irradiation to the cell’s basal membrane^13^. Therefore, phototoxic effects are reduced when irradiation is performed in TIRF mode^19^.

Recently, the question how much light biological specimen can withstand was addressed by Ernst Stelzer who suggested the solar constant (~100 mW cm^−2^, central Europe) as a reference^32^. After 600 s sunlight, the energy density is about 0.6 μJ μm^−2^ (60 J cm^−2^). Albeit half of the sun’s irradiation energy is in the infrared spectral range, the estimated value is in general accordance with our result of a maximum dose of 48 J cm^−2^ at 405 nm irradiation (Fig. 3b, Supplementary Table 2) and the result reported by Wagner *et al.* of ~25 J cm^−2^ at 375 nm^19^.

However, our experiments using pulsed irradiation (Fig. 3b) reveal that the irradiation dose alone does not determine the degree of photodamage. For a total irradiation time of 24 s at 405 nm with an intensity of 0.02 kW cm^−2^ our data unravel that substantially less U2OS cells survive pulsed irradiation than cw irradiation albeit they experienced the same light dose of 480 J cm^−2^ (Fig. 3b). On the other hand, all cells survive pulsed irradiation at 405 nm with pulse lengths of 1 ms at 10 Hz applied for a short irradiation time of 2.4 s (Fig. 3b). This indicates that cells can cope with the concentration of phototoxic molecules generated during 2,400 1 ms irradiation periods. For longer irradiation periods and irradiation times more toxic molecules are generated surpassing the cells’ repair capacity.

Whether our results can be applied to other fluorescence and super-resolution microscopy methods remains to be experimentally verified but can be carefully estimated. Confocal laser scanning microscopy operates typically at irradiation intensities of 10–50 kW cm^−2^ yet focuses the energy on a very small spot and uses short irradiation times typically in the range of 10–500 μs per pixel and frame^33^. Using these values and a pixel size of ~120 nm we estimate that cells experience a light dose of less than 500 J cm^−2^ per image. That is, according to our studies, cells should survive confocal laser scanning microscopy at irradiation wavelengths of ≥488 nm as long as the total light dose experienced does not exceed a few kJ cm^−2^ (Fig. 3a and Supplementary Tables 1 and 2). On the other hand, it is very likely that live-cell laser scanning microscopy at irradiation wavelengths <488 nm causes severe photodamage.

Stimulated emission depletion (STED) microscopy uses an additional depletion laser with an irradiation wavelength usually in the red to near-infrared spectral region (typically at 647 or 800 nm), i.e., in a wavelength range where cells survive irradiation intensities of kW cm^−2^ applied for a few minutes (Fig. 3a). However, the irradiation intensity of the depletion laser is typically three to five orders of magnitude higher than the excitation laser because the resolution of STED microscopy scales with the depletion laser intensity^34^,^35^. Thus, the total light dose impinging on the cells might easily approach 10^4^–10^6^ kJ cm^−2^ and potentially induce photodamage effects even by irradiation with wavelengths >600 nm (Fig. 3a and Supplementary Table 1).

RESOLFT nanoscopy uses photoswitchable fluorescent proteins such as rsEGFP^36^ and Dreiklang^37^ as photoswitches. Hence, the applied laser power for activation, readout and photoswitching are reduced to 0.1–10 kW cm^−2^, but lasers in the (ultra)violet and blue spectral range are required, e.g., Dreiklang at wavelengths of 355 nm for activation (0.3 kW cm^−2^, 1 ms), 405 nm for photoswitching (10 kW cm^−2^, 17 ms), and 491 nm for readout (0.8 kW cm^−2^, 2 ms) (Fig. 3a)^38^. Thus, the corresponding light doses exceed the 48 J cm^−2^ level determined in our study for 405 nm irradiation and induce photodamage (Fig. 3b, Supplementary Table 2). A way out of this problem offer new improved photoswitchable fluorescent proteins such as rsEGFP2, which require a light dose of only 2–10 J cm^−2^ for photoswitching at 405 nm^39^, and thus enable photodamage-free super-resolution imaging over longer time periods.

To minimize photodamage problems induced by high peak irradiation intensities in confocal live-cell fluorescence microscopy schemes, light-sheet microscopy is usually applied enabling fascinating studies about the development of living organism also over longer time periods at cellular level^1^,^3^. To achieve subcellular resolution, lattice light-sheet microscopy in combination with structured illumination has been developed^4^. The method uses ultrathin light sheets which are scanned plane-by-plane through the specimen to generate a 3D image. The laser power of 1–100 μW^4^ used for excitation of living cells and organisms is similar to confocal microscopy but distributed over substantially larger areas in the focal plane. Therefore, the light dose impinging on the cells is substantially lower than in confocal microscopy approaches and accordingly light-induced cell damage significantly reduced.

Generally, it is assumed that the resolution of wide-field super-resolution microscopy methods comes along with the irradiation intensity. For example, single-molecule localization microscopy achieves typically a lateral resolution of ~20 nm^5^,^6^,^7^,^8^ but requires naturally also higher irradiation intensities (1–5 kW cm^−2^) than methods which achieve a lower spatial resolution, e.g., structured illumination microscopy (SIM)^40^. However, our data clearly demonstrate that living cells can tolerate such high irradiation intensities if excitation is performed at 640 nm or longer wavelengths (Fig. 3a).

As we have seen that HeLa cells withstand much higher irradiation intensities than U2OS cells (Fig. 4) it remains to be tested whether all findings can be directly transferred to other cell lines or living organisms. In addition, cell cycle dependent mechanisms have to be considered. Even within the same population individual cells might respond differently to excessive irradiation^41^. This also might explain fluctuations in the photodamage data found in our study (Fig. 2 and Fig. 4).

Nevertheless, our results give important advice how to perform live-cell super-resolution microscopy experiments (Table 2). The use of switching buffers that include enzymatic oxygen scavenging systems and thiol containing reducing agents will add additional stress to cells in live cell single-molecule localization microscopy experiments (Fig. 6) and should therefore be avoided. Since living cells contain the thiol glutathione at millimolar concentration levels as native reducing agent live-cell *d*STORM in standard media is possible with selected organic fluorophores without addition of external thiols^11^,^14^,^24^,^42^,^43^,^44^. Strategies to maintain cell health can be either achieved by reducing the illumination depth through TIRF or single-plane illumination^1^, or increasing the photodamage resistance, e.g., by imaging at 37 °C and the addition of μM concentrations of ascorbic acid (Table 2). At the same time, ascorbic acid could also serve as photoswitching reagent^45^. On the other hand, phototoxic effects at high irradiation intensities can be substantially reduced when exciting fluorophores at >600 nm, motivating the development of new (near infra-) red absorbing photoswitchable FPs and live-cell compatible organic dyes^46^,^47^,^48^. Independent of the method and the wavelength used, live-cell super-resolution microscopy experiments require stringent tests to verify that the cellular processes observed are not influenced by the high irradiation intensities.

## Online Materials and Methods

### Cell culture

All cell lines were cultured at 37 °C and 5% CO_2_. U2OS cells (human osteosarcoma cell line) and COS-7 cells (monkey kidney fibroblast cell line) were grown in DMEM F12 with L-glutamine (Sigma, cat. D8062) supplemented with 10% FBS (Sigma, cat. F7524) and 1% penicillin-streptomycin (Sigma, cat. P4333). HeLa cells (human cervical adenocarcinoma cell line) were cultured in RPMI-1640 medium with L-glutamine (Sigma, cat. R8758) supplemented with 1% MEM Non-essential Amino Acid Solution (Sigma, cat. M7145), 1 mM sodium pyruvate (Sigma, cat. S8636), 10% FBS (Sigma, cat. F7524) and 1% penicillin-streptomycin (Sigma, cat. P4333). All cell lines have been passaged more than 25 times before experiments. Live-cell imaging and irradiation were performed in medium without phenol red: DMEM F12 with 15 mM HEPES (Sigma, cat. D6434) and RPMI-1640 medium (Sigma, cat. R7509). The U2OS CLIP-H2B cell line stably expressing CLIP-H2B was transfected using the transfection reagent FuGENE® HD (promega, cat. E2311) and selected by 0.3 mg/ml G418 (Sigma, cat. G8168). Cells were stained with 0.2 μM CLIP-Cell™ TMR-Star (New England Biolabs, cat. S9219S) for 30 minutes at 37 °C. Afterwards cells were washed three times with medium. Ascorbic acid (100 μM) was added one hour before the experiments. After irradiation, the AA buffer was replaced by standard medium.

### *d*STORM

Irradiation and imaging were performed on a widefield setup^42^. As laser light sources we used 405 and 488 nm diode lasers (iBeam smart Family, TOPTICA Photonics) with 120 mW and 200 mW total output power, respectively, as well as three optically pumped semiconductor lasers (OPSL, Genesis MX STM-Series, Coherent), i.e., 514 nm, 558 nm and 640 nm with 500, 500, and 1000 mW total output power, respectively. Laser beams were cleaned-up by bandpass filters (Semrock/Chroma) and combined by appropriate dichroic mirrors (LaserMUX, Semrock). Afterwards, they were focused onto the back focal plane of a high numerical oil-immersion objective (Olympus APON 60XO TIRF, NA 1.49), which is part of an inverted fluorescence microscope (Olympus IX71). To separate the excitation light from the fluorescence light, suitable dichroic beam splitters (Semrock) were placed into the light path before the laser beams enter the objective. Fluorescence light collected by the objective was filtered by appropriate detection filters (Semrock/Chroma) and imaged with additional optical magnification by an EMCCD camera with 512 × 512 pixels (iXon Ultra 897, Andor Technology). The achieved pixel size was 128 nm/px.

### Irradiation and determination of photodamage

For irradiation experiments cells were seeded in petri dishes with an imprinted 500 μm relocation grid (ibidi, cat. 81168) one or two days before the experiment. Cells were irradiated with the desired wavelength at 21 °C unless otherwise stated, a defined laser intensity and for a particular time (240 s unless stated otherwise) in EPI or in HILO mode. The experiments duration was kept constant at 2.5 hours. Using a rectangular field stop (OWIS) irradiation was restricted to cells present in the defined field of view of 65.5 μm × 65.5 μm. To achieve virtually homogeneous irradiation over the entire field of view the laser beams were largely expanded. The irradiation intensity varied between 6 and 12% from the center to the edge of the field of view (Supplementary Fig. 7). The laser power was measured above the objective using a laser power meter (LabMax-TO, Coherent). The illuminated intensity in HILO mode was ascertained as described by Tokunaga *et al.*^10^. According to errors in the adjustment of the field stop, the determination of the incidence angle and the measurement of the laser power we assume an uncertainty in intensity assignment of about 5–6%. For experiments at 37 °C a custom build incubation chamber was used, which was put on the x-y stage of the microscope.

After illumination of several cells, the medium was changed against fresh medium and irradiated cells were observed in a live cell recorder (BioStation IM, Nikon) for the following 20–24 hours (0.2 images min^−1^). The recorded images were assembled to an image stack for cell damage analysis.

### Microtubule and actin staining

Cells of the stable U2OS CLIP-H2B cell line were seeded in petri dishes with an imprinted 500 μm relocation grid (ibidi, cat. 81168) one or two days before the experiment. They were irradiated at 405 nm for 240 s at 37 °C with an intensity of 0.05 kW/cm^2^ for microtubule staining and 0.24 kW cm^−2^ for actin staining. Microtubules were stained with 1 μM SiR-taxol^44^ in medium for 30 minutes at 37 °C. Actin was stained with 33 nM Alexa Fluor 647 phalloidin (Molecular Probes, cat. A22287) in PBS for 30 minutes at room temperature. Cells were washed with PBS and imaged at 640 nm using a 679/41 single-band bandpass filter (Semrock). For *d*STORM imaging phosphate buffered saline (pH 7.4) containing the following components was used as switching buffer: 100 mM β-mercaptoethylamine (AppliChem, cat. A1546), 4% glucose (w/v) (Merck, cat. 108337), 10 U ml^−1^ glucose oxidase (Sigma, cat. G2133), 200 U ml^−1^ catalase (Sigma, cat. C100)^49^.

### Microtubule tracking

The stable cell line HeLa EB1-N-YFP was given to us by the Medical University of Innsbruck (Molecular Pathophysiology, Prof. Dr. Stefan Geley). All measurements were conducted at the setup described above at 37 °C. MT-growth was recorded for 50 s by exciting EB1-N-YFP with 488 nm at minimal intensities (<10 W cm^−2^) within the HiLO mode before and after irradiation of the cell. In these measurements, the 488 nm laser and the EMCCD camera were synchronized by a pulse generator (DG645, Stanford Research Systems, Gilching, Germany), i.e., 2 Hz pulse frequency and 100 ms integration time to minimize irradiation effects. Cells were irradiated for 225 s with 558 nm and 640 nm laser excitation in the Epi mode with different intensities, respectively (0–4.48 kW cm^−2^ and 0–10.09 kW cm^−2^). Tracking of the EB1-spots and computation of the microtubule growth speed were performed using the software Imaris (Bitplane, Zurich, Switzerland). The median of the MT-growth speed before and after irradiation were subtracted and the percentage of deceleration calculated.

## Additional Information

**How to cite this article**: Wäldchen, S. *et al.* Light-induced cell damage in live-cell super-resolution microscopy. *Sci. Rep.* **5**, 15348; doi: 10.1038/srep15348 (2015).

## Supplementary Material

Supplementary Video 1

Supplementary Video 2

Supplementary Video 3

Supplementary Information

## Figures and Tables

**Table 1:** Effects of intracellular and environmental conditions on photo-sensitivity of cells.

	Conditions	*I*_50_ (kW cm^−2^)^a^		LD50 (kJ cm^−2^)		Number of cells	
		‘dead’	‘frozen’	‘dead’	‘frozen’	total	per datapoint^b^
U2OS	untreated	0.43 ± 0.01	1.06 ± 0.02	104.4	254.1	404	36.7 ± 19.2
	transfected	0.34 ± 0.02	1.31 ± 0.06	81.6	313.9	707	22.1 ± 17.7
	transfected+TMR	0.24 ± 0.01	1.38 ± 0.03	58.4	331.6	599	20.7 ± 10.8
	transfected+37 °C	0.58 ± 0.05	1.06 ± 0.03	138.4	253.6	385	42.8 ± 14.1
	transfected+AA	0.54 ± 0.06	n.d.	129.6	n.d.	458	28.6 ± 21.7
COS-7	untreated	0.53 ± 0.01	0.88 ± 0.04	126.5	211.6	239	26.6 ± 3.5
HeLa	untreated	2.82 ± 0.62	n.d.	677.5	n.d.	256	32.0 ± 8.8

If not otherwise stated, cells were irradiated at 21 °C. I_50_ value is the intensity where 50% of the irradiated cells died after imaging at 514 nm for 240 s. U2OS cells were transfected with CLIB-H2B and optionally imaged with TMR, at 37 °C, or supplemented with 100 μM ascorbic acid (AA).

^a^Errors are standard errors from data fits.

^b^Errors are given as one standard deviation. n.d., not determined.

**Table 2:** Recommendations for live-cell single-molecule localization microscopy experiments.

Parameter	Recommendation	Example
Wavelength	High	640 nm
Intensity	Low	<6 kW cm^−2^ (640 nm)
Activation	No	ATTO 655*
Cell type	Photo-insensitive	HeLa
Illumination type	Reduced penetration depth	TIRF
Temperature	Physiological	37 °C
Switching buffer	No	cell culture medium
Protecting agents	Depending	AA

AA, ascorbic acid.

^*^Ref. 14.

## References

[b1] Huisken J., Swoger J., Del Bene F., Wittbrodt J. & Stelzer E. H. Optical sectioning deep inside live embryos by selective plane illumination microscopy. Science 305, 1007–1009 (2004). 15310904 10.1126/science.1100035

[b2] Gao L. *et al.* Noninvasive imaging beyond the diffraction limit of 3D dynamics in thickly fluorescent specimens. Cell 151, 1370–1385 (2012). 23217717 10.1016/j.cell.2012.10.008 PMC3615549

[b3] Keller P. J. *et al.* Fast, high-contrast imaging of animal development with scanned light sheet-based structured-illumination microscopy. Nat. Methods 7, 637–642 (2010). 20601950 10.1038/nmeth.1476 PMC4418465

[b4] Chen B. C. *et al.* Lattice light-sheet microscopy: imaging molecules to embryos at high spatiotemporal resolution. Science 346, 1257998 (2014). 25342811 10.1126/science.1257998 PMC4336192

[b5] Betzig E. *et al.* Imaging intracellular fluorescent proteins at nanometer resolution. Science 313, 1642–1645 (2006). 16902090 10.1126/science.1127344

[b6] Hess S. T., Girirajan T. P. & Mason M. D. Ultra-high resolution imaging by fluorescence photoactivation localization microscopy. Biophys. J. 91, 4258–4272 (2006). 16980368 10.1529/biophysj.106.091116 PMC1635685

[b7] Rust M. J., Bates M. & Zhuang X. W. Sub-diffraction-limit imaging by stochastic optical reconstruction microscopy (STORM). Nat. Methods 3, 793–795 (2006). 16896339 10.1038/nmeth929 PMC2700296

[b8] Heilemann M. *et al.* Subdiffraction-resolution fluorescence imaging with conventional fluorescent probes. Angew. Chem. Int. Ed. Engl. 47, 6172–6176 (2008). 18646237 10.1002/anie.200802376

[b9] van de Linde S. & Sauer M. How to switch a fluorophore: from undesired blinking to controlled photoswitching. Chem. Soc. Rev. 43, 1076–1087 (2014). 23942584 10.1039/c3cs60195a

[b10] Tokunaga M., Imamoto N. & Sakata-Sogawa K. Highly inclined thin illumination enables clear single-molecule imaging in cells. Nat. Methods 5, 159–161 (2008). 18176568 10.1038/nmeth1171

[b11] van de Linde S., Heilemann M. & Sauer M. Live-cell super-resolution imaging with synthetic fluorophores. Annu. Rev. Phys. Chem. 63, 519–540 (2012). 22404589 10.1146/annurev-physchem-032811-112012

[b12] Editorial: Artifacts of light. Nat. Methods 10, 1135 (2013).

[b13] Shroff H., Galbraith C. G., Galbraith J. A. & Betzig E. Live-cell photoactivated localization microscopy of nanoscale adhesion dynamics. Nat. Methods 5, 417–423 (2008). 18408726 10.1038/nmeth.1202 PMC5225950

[b14] Wombacher R. *et al.* Live-cell super-resolution imaging with trimethoprim conjugates. Nat. Methods 7, 717–719 (2010). 20693998 10.1038/nmeth.1489

[b15] Jones S. A., Shim S. H., He J. & Zhuang X. Fast, three-dimensional super-resolution imaging of live cells. Nat. Methods 8, 499–505 (2011). 21552254 10.1038/nmeth.1605 PMC3137767

[b16] Carlton P. M. *et al.* Fast live simultaneous multiwavelength four-dimensional optical microscopy. Proc. Natl. Acad. Sci. USA 107, 16016–16022 (2010). 20705899 10.1073/pnas.1004037107 PMC2941331

[b17] Khodjakov A. & Rieder C. L. Imaging the division process in living tissue culture cells. Methods 38, 2–16 (2006). 16343936 10.1016/j.ymeth.2005.07.007 PMC2590767

[b18] Manders E. M. M., Kimura H. & Cook P. R. Direct imaging of DNA in living cells reveals the dynamics of chromosome formation. J. Cell Biol. 144, 813–821 (1999). 10085283 10.1083/jcb.144.5.813 PMC2148202

[b19] Wagner M. *et al.* Light Dose is a Limiting Factor to Maintain Cell Viability in Fluorescence Microscopy and Single Molecule Detection. Int. J. Mol. Sci. 11, 956–966 (2010). 20479994 10.3390/ijms11030956 PMC2869222

[b20] Berridge M. V., Herst P. M. & Tan A. S. Tetrazolium dyes as tools in cell biology: new insights into their cellular reduction. Biotechnol. Annu. Rev. 11, 127–152 (2005). 16216776 10.1016/S1387-2656(05)11004-7

[b21] Riss T. L. *et al.*. In Assay Guidance Manual. (eds. Sittampalam G. S. *et al.*) (Bethesda (MD); 2004).

[b22] Mosmann T. Rapid colorimetric assay for cellular growth and survival: application to proliferation and cytotoxicity assays. J. Immunol. Methods 65, 55–63 (1983). 6606682 10.1016/0022-1759(83)90303-4

[b23] Berridge M. V. & Tan A. S. Characterization of the cellular reduction of 3-(4,5-dimethylthiazol-2-yl)-2,5-diphenyltetrazolium bromide (MTT): subcellular localization, substrate dependence, and involvement of mitochondrial electron transport in MTT reduction. Arch. Biochem. Biophys. 303, 474–482 (1993). 8390225 10.1006/abbi.1993.1311

[b24] Klein T. *et al.* Live-cell dSTORM with SNAP-tag fusion proteins. Nat. Methods 8, 7–9 (2011). 21191367 10.1038/nmeth0111-7b

[b25] Schuyler S. C. & Pellman D. Microtubule “plus-end-tracking proteins”: The end is just the beginning. Cell 105, 421–424 (2001). 11371339 10.1016/s0092-8674(01)00364-6

[b26] Vitre B. *et al.* EB1 regulates microtubule dynamics and tubulin sheet closure *in vitro*. Nat. Cell Biol. 10, 415–421 (2008). 18364701 10.1038/ncb1703

[b27] Akhmanova A. & Steinmetz M. O. Microtubule plus TIPs at a glance. J. Cell Sci. 123, 3415–3419 (2010). 20930136 10.1242/jcs.062414

[b28] Ochsner M. Photophysical and photobiological processes in the photodynamic therapy of tumours. J. Photochem. Photobiol. B 39, 1–18 (1997). 9210318 10.1016/s1011-1344(96)07428-3

[b29] Castano A. P., Mroz P. & Hamblin M. R. Photodynamic therapy and anti-tumour immunity. Nat. Rev. Cancer 6, 535–545 (2006). 16794636 10.1038/nrc1894 PMC2933780

[b30] Coohill T. P., Peak M. J. & Peak J. G. The effects of the ultraviolet wavelengths of radiation present in sunlight on human cells *in vitro*. Photochem. Photobiol. 46, 1043–1050 (1987). 3325999 10.1111/j.1751-1097.1987.tb04891.x

[b31] Izeddin I. *et al.* Super-Resolution Dynamic Imaging of Dendritic Spines Using a Low-Affinity Photoconvertible Actin Probe. PLoS ONE 6 e15611 (2011). 21264214 10.1371/journal.pone.0015611 PMC3022016

[b32] Stelzer E. H. K. Light-sheet fluorescence microscopy for quantitative biology. Nat. Methods 12, 23–26 (2014). 25549266 10.1038/nmeth.3219

[b33] Pawley J. B. Handbook of Biological Confocal Microscopy. (Springer, Heidelberg; 2006).

[b34] Hein B., Willig K. I. & Hell S. W. Stimulated emission depletion (STED) nanoscopy of a fluorescent protein-labeled organelle inside a living cell. Proc. Natl. Acad. Sci. USA 105, 14271–14276 (2008). 18796604 10.1073/pnas.0807705105 PMC2538451

[b35] Hein B. *et al.* Stimulated emission depletion nanoscopy of living cells using SNAP-tag fusion proteins. Biophys. J. 98, 158–163 (2010). 20074516 10.1016/j.bpj.2009.09.053 PMC2800968

[b36] Grotjohann T. *et al.* Diffraction-unlimited all-optical imaging and writing with a photochromic GFP. Nature 478, 204–208 (2011). 21909116 10.1038/nature10497

[b37] Brakemann T. *et al.* A reversibly photoswitchable GFP-like protein with fluorescence excitation decoupled from switching. Nat. Biotechnol. 29, 942–947 (2011). 21909082 10.1038/nbt.1952

[b38] Jensen N. A. *et al.* Coordinate-targeted and coordinate-stochastic super-resolution microscopy with the reversibly switchable fluorescent protein Dreiklang. ChemPhysChem 15, 756–762 (2014). 24497300 10.1002/cphc.201301034

[b39] Grotjohann T. *et al.* rsEGFP2 enables fast RESOLFT nanoscopy of living cells. eLife 1, e00248 (2012). 23330067 10.7554/eLife.00248 PMC3534202

[b40] Shao L., Kner P., Rego E. H. & Gustafsson M. G. L. Super-resolution 3D microscopy of live whole cells using structured illumination. Nat. Methods 8, 1044-+ (2011). 22002026 10.1038/nmeth.1734

[b41] Magidson V. & Khodjakov A. Circumventing photodamage in live-cell microscopy. Methods Cell Biol. 114, 545–560 (2013). 23931522 10.1016/B978-0-12-407761-4.00023-3 PMC3843244

[b42] van de Linde S. *et al.* Direct stochastic optical reconstruction microscopy with standard fluorescent probes. Nat. Protoc. 6, 991–1009 (2011). 21720313 10.1038/nprot.2011.336

[b43] Shim S. H. *et al.* Super-resolution fluorescence imaging of organelles in live cells with photoswitchable membrane probes. Proc. Natl. Acad. Sci. USA. 109, 13978–13983 (2012). 22891300 10.1073/pnas.1201882109 PMC3435176

[b44] Carlini L. & Manley S. Live intracellular super-resolution imaging using site-specific stains. ACS Chem. Biol. 8, 2643–2648 (2013). 24079385 10.1021/cb400467x

[b45] Vogelsang J., Cordes T., Forthmann C., Steinhauer C. & Tinnefeld P. Controlling the fluorescence of ordinary oxazine dyes for single-molecule switching and superresolution microscopy. Proc. Natl. Acad. Sci. USA. 106, 8107–8112 (2009). 19433792 10.1073/pnas.0811875106 PMC2688868

[b46] Lukinavicius G. *et al.* Fluorogenic probes for live-cell imaging of the cytoskeleton. Nat. Methods 11, 731–U168 (2014). 24859753 10.1038/nmeth.2972

[b47] Grimm J. B. *et al.* A general method to improve fluorophores for live-cell and single-molecule microscopy. Nat. Methods 12, 244–250 (2015). 25599551 10.1038/nmeth.3256 PMC4344395

[b48] Uno S. N. *et al.* A spontaneously blinking fluorophore based on intramolecular spirocyclization for live-cell super-resolution imaging. Nat. Chem. 6, 681–689 (2014). 25054937 10.1038/nchem.2002

[b49] Schafer P., van de Linde S., Lehmann J., Sauer M. & Doose S. Methylene blue- and thiol-based oxygen depletion for super-resolution imaging. Anal. Chem. 85, 3393–3400 (2013). 23410003 10.1021/ac400035k

