# Supplementary material for: Light-induced cell damage in live-cell super-resolution microscopy
Source: Sci Rep. 2015 Oct 20;5:15348. doi: 10.1038/srep15348 (PMC4611486; doi:10.1038/srep15348)
Supplement: Supplementary Information [file srep15348-s4.pdf]

Supplementary Information

Light-induced cell damage in live-cell super-resolution microscopy

Sina Wäldchen, Julian Lehmann,
Teresa Klein, Sebastian van de Linde, Markus Sauer

Department of Biotechnology and Biophysics, Biocenter, University of Würzburg, Am Hubland, 97074 Würzburg, Germany

Supplementary Table 1. Wavelength and intensity dependence on cell health. 240 s irradiation time. [a] Errors are given as one standard deviation.

Wavelength (nm)	Intensity (kW cm^{-2})	Light dose (kJ cm^{-2})	Fraction (%) [a]		Number of cells
			dead	frozen	
405	0.023	5.52	100	100	38
405	0.187	44.85	100	100	21
488	0.187	44.85	100	41 ± 41	19
514	0.193	46.38	0	0	9
488	0.776	186.31	100	100	21
514	0.791	189.77	100	17 ± 29	10
514	2.013	483.04	100	92 ± 17	10
558	2.013	483.04	17 ± 32	0	32
640	2.013	483.04	0	0	34
640	4.025	966.07	6 ± 13	0	36
640	5.894	1,414.61	2 ± 6	0	37

Supplementary Table 2. Effect of pulsed irradiation on cell health. 405 nm, 0.02 kW cm^{-2}. [a] Errors are given as one standard deviation.

Irradiation time (s)	Pulse frequency (Hz)	Pulse length (s)	Total acquisition time (s)	Light dose (kJ cm^{-2})	Fraction (%) [a]		Number of cells
					dead	frozen	
2.4	10	0.001	240	0.048	0	0	28
	5	0.002	240	0.048	4 ± 12	0	30
	1	0.01	240	0.048	3 ± 8	0	36
12	5	0.01	240	0.24	69 ± 21	0	29
24	10	0.01	240	0.48	98 ± 8	3 ± 8	30
	5	0.02	240	0.48	97 ± 11	0	33
	1	0.1	240	0.48	90 ± 25	0	23
	cw	cw	24	0.48	14 ± 20	0	25
60	5	0.05	240	1.2	100	81 ± 23	25
	cw	cw	60	1.2	100	17 ± 41	18
120	1	0.5	240	2.4	100	93 ± 17	31
	cw	cw	120	2.4	100	79 ± 29	27

Supplementary Table 3. Microtubule growth speed of single cells before and after irradiation.

Irradiation wavelength [nm]	Intensity [kW cm^{-2}]	Percentage of deceleration [%]	Growth speed (median) [μm min^{-1}]		Number of tracks	
			before	after	before	after
No additional irradiation	-	14	7.2	6.5	443	463
		9	4.7	4.3	265	161
		11	4.6	4.1	476	379
		24	4.6	3.5	302	299
		18	5.5	4.5	451	519
		10	6.1	5.5	478	511
		13	5.9	5.1	497	463
		-1	11.3	11.4	285	353
		-2	9.6	9.8	447	482
		22	9.3	7.2	336	285
		4	9.2	8.8	138	133
		13	8.4	7.3	298	268
		2	8.8	8.6	229	198
		3	6.1	5.9	112	127
558	0.43	65	9.7	3.4	491	282
		71	9.4	2.8	421	226
		58	7.7	3.2	551	558
		83	8.2	1.4	665	32
	0.91	65	3.7	1.3	757	446
		73	6.5	1.8	427	309
		73	5.7	1.6	526	120
		77	8.1	1.8	707	365
		68	4.6	1.5	284	234
		65	6.7	2.3	487	148
		79	6.6	1.4	310	17
	1.4	75	7.0	1.8	668	216
		71	7.3	2.1	232	7
		77	8.3	1.9	648	15
	1.88	77	5.9	1.3	407	11
640	0.03	12	5.1	4.5	600	425
		39	9.5	5.8	306	199
	0.07	25	5.4	4.0	389	340
		35	8.6	5.5	136	50
	0.16	53	8.1	3.8	317	167
		19	12.0	9.8	226	15
	0.43	58	4.4	1.8	331	70
		56	6.4	2.8	363	279
		33	8.4	5.6	367	405
		43	10.3	5.9	200	195
	0.88	83	4.3	0.7	272	1
		27	3.5	2.5	224	313
		48	13.2	6.8	169	27
	2.39	57	8.0	3.5	140	116
		21	8.0	6.3	129	89
		50	7.3	3.6	151	131
	4.96	44	5.1	2.9	140	67
		37	8.9	5.6	134	88
		50	8.4	4.2	195	172
		46	8.4	4.5	193	34
	10.09	66	8.9	3.1	164	67

42	6.6	3.9	175	109
44	6.5	3.6	223	154
41	7.1	4.2	224	83

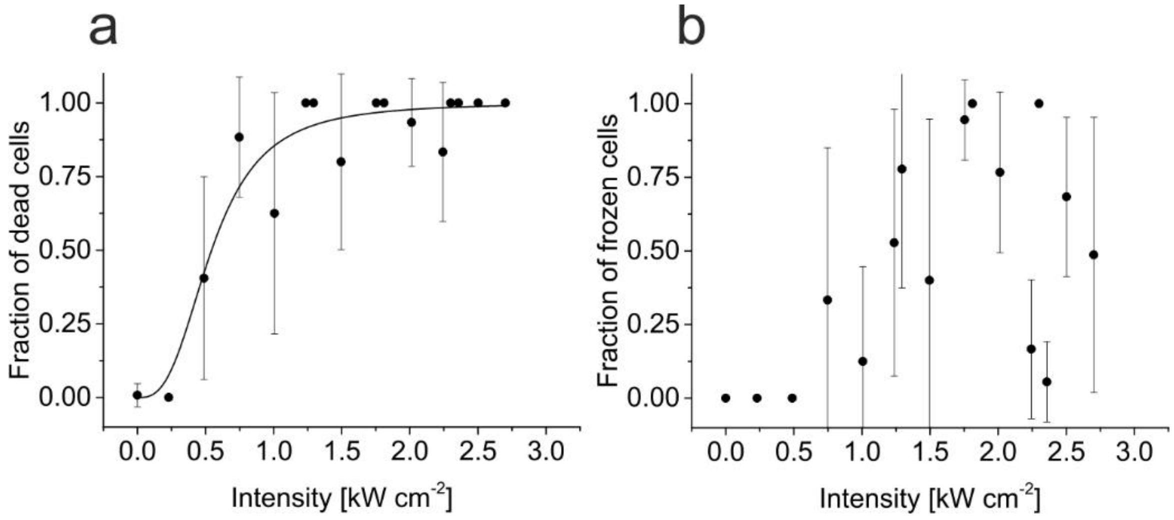

Supplementary Figure 1. Dependence of cell survival on irradiation intensity with 100 µM ascorbic acid as cell medium supplement. **a**) Dead cells, **b**) frozen cells. For each data point 20-50 cells were irradiated (**Table 1)**.

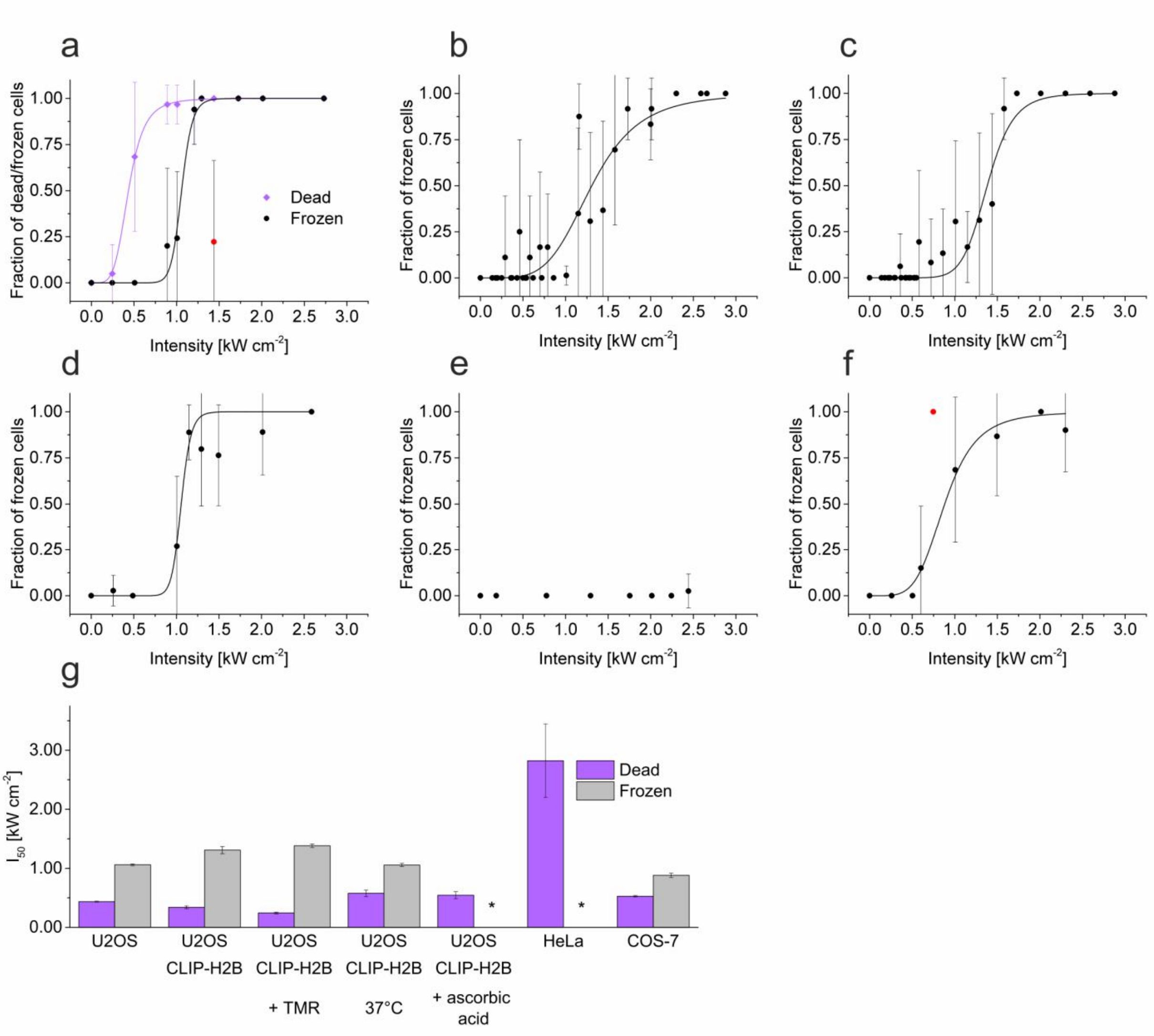

Supplementary Figure 2. Dependence of cell survival on irradiation intensity. Data were modeled with unweighted logistic fits. (**a-d**) Fraction of frozen U2OS cells; **a**) wildtype (dead and frozen), **b**) stably transfected, **c**) stained, **d**) 37°C. Fraction of frozen **e**) HeLa cells and **f**) COS-7. (a-f) Error bars are given as one standard deviation. For each data point 20-50 cells were irradiated (**Table 1)**. **g**) i_{50} values for dead and frozen cells. Errors are standard errors of data fits.

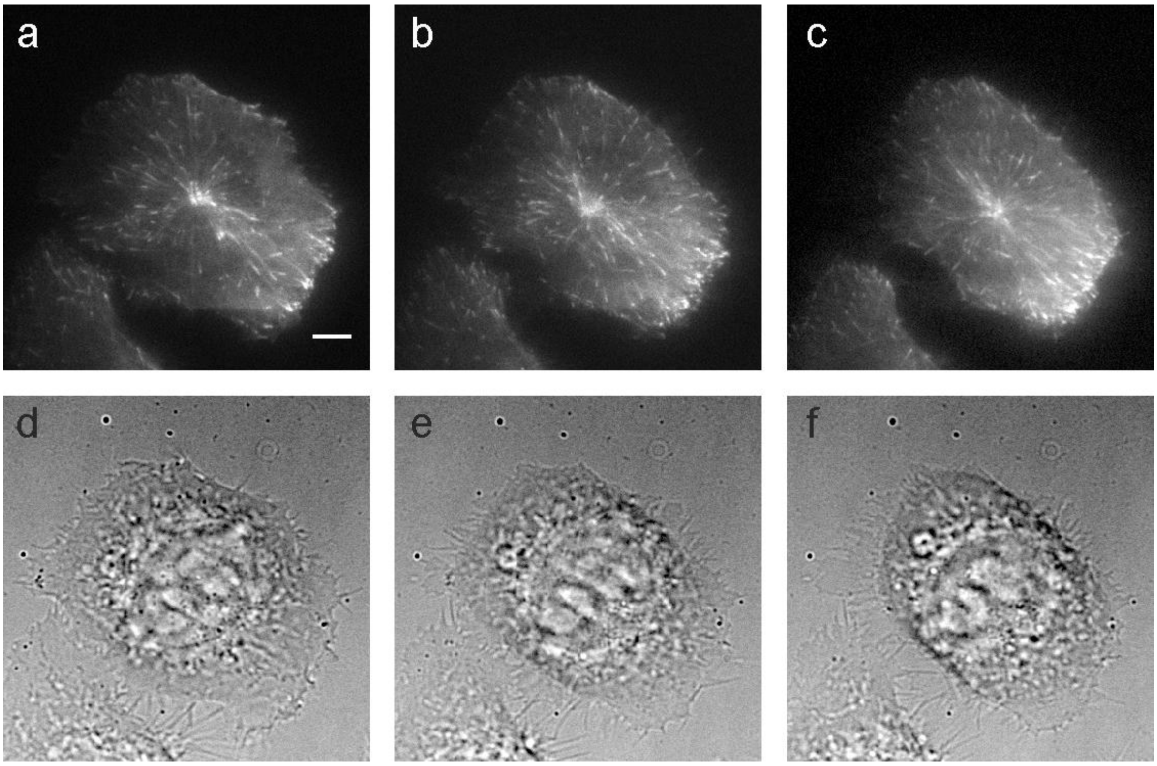

Supplementary Figure 3. EB1 measurements of a cell without additional irradiation. (**a-c**) Fluorescence images and (**d-f**) corresponding bright field images. (**a, d**) Initial EB1-N-YFP fluorescence showing an adherent and vital cell. (**b, e**) EB1-YFP after microtubule growth measurements. Cells were first irradiated for 50 s at 488 nm with < 10 W cm^{-2} (2 Hz, 100 ms integration time), next kept in the dark for period of 225 s without additional irradiation followed by a second microtubule growth measurement for 50 s. MT-growth shows only a slight deceleration and no abnormal morphological changes of the cell. (**c, f**) 5 min after (**b**, **e**) showing no obvious photodamage effects (cf. **Supplementary Figure 4**). Measurements were done at 37°C. Scale bar, 5 µm.

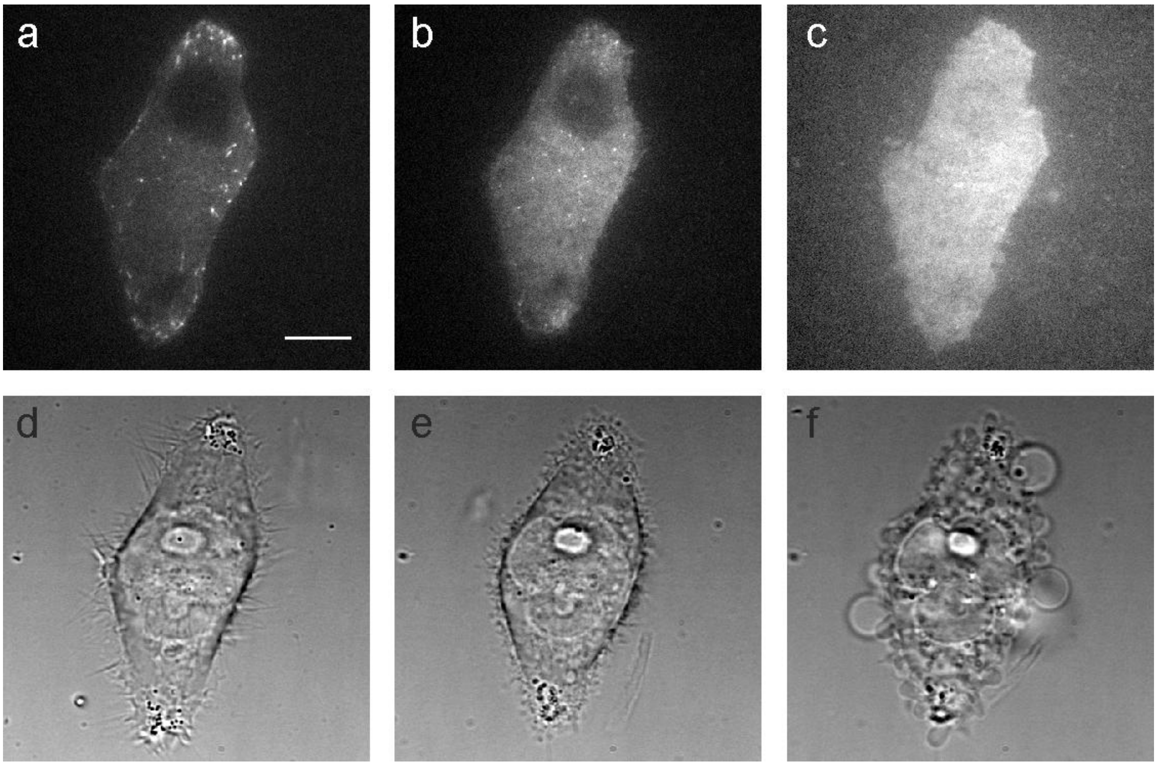

Supplementary Figure 4. EB1 measurements of a cell with additional irradiation at 558 nm. (**a-c**) Fluorescence images and (**d-f**) corresponding bright field images. (**a, d**) Initial EB1-N-YFP fluorescence showing an adherent and vital cell. (**b, e**) EB1-N-YFP after microtubule (MT) growth measurements and additional irradiation. Cells were first irradiated for 50 s at 488 nm with < 10 W cm^{-2} (2 Hz, 100 ms integration time), next irradiated at 558 nm with 0.91 kW cm^{-2} for 225 s followed by a second microtubule growth measurement for 50 s. **b**) Slow MT- growth is still recordable (some bright dots), but MT structure seems to be highly damaged (bright unstructured background). **e**) Changes of the cell membrane (loss of filopodia). (**c, f**) 5 min after (**b**, **e**) showing total loss of the MT-structure (only unstructured YFP fluorescence). **f**) Cell membrane breakdown and cytosol leakage. Measurements were done at 37°C. Scale bar, 10 μm.

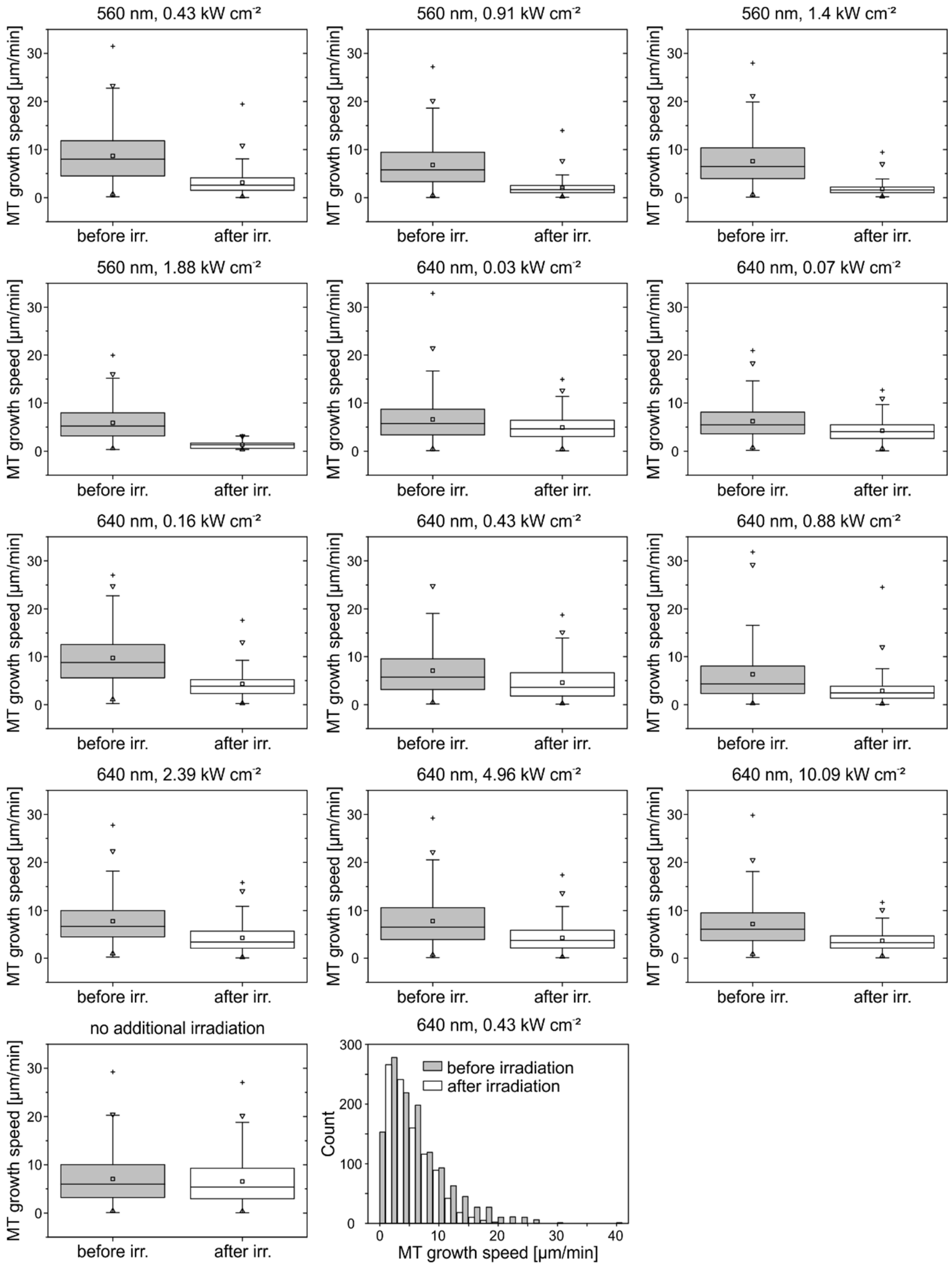

Supplementary Figure 5. Boxplots of datasets of microtubule growth speed analysis before and after irradiation with different wavelengths and intensities. The number of tracks analyzed per boxplot is given in **Supplementary Table 3.** The difference in percent between the median (horizontal lines) before and after irradiation is plotted in **Fig. 6b**. Whiskers span the range of 1.5 IQR, □ indicates the mean, △ marks 1%, ▽ 99% and + the maximum of all data points. The histogram of MT-growth speed before and after irradiation (example dataset 640 nm, 0.43 kW cm^{-2}) shows a non-normal distribution; therefore medians were used for further analysis.

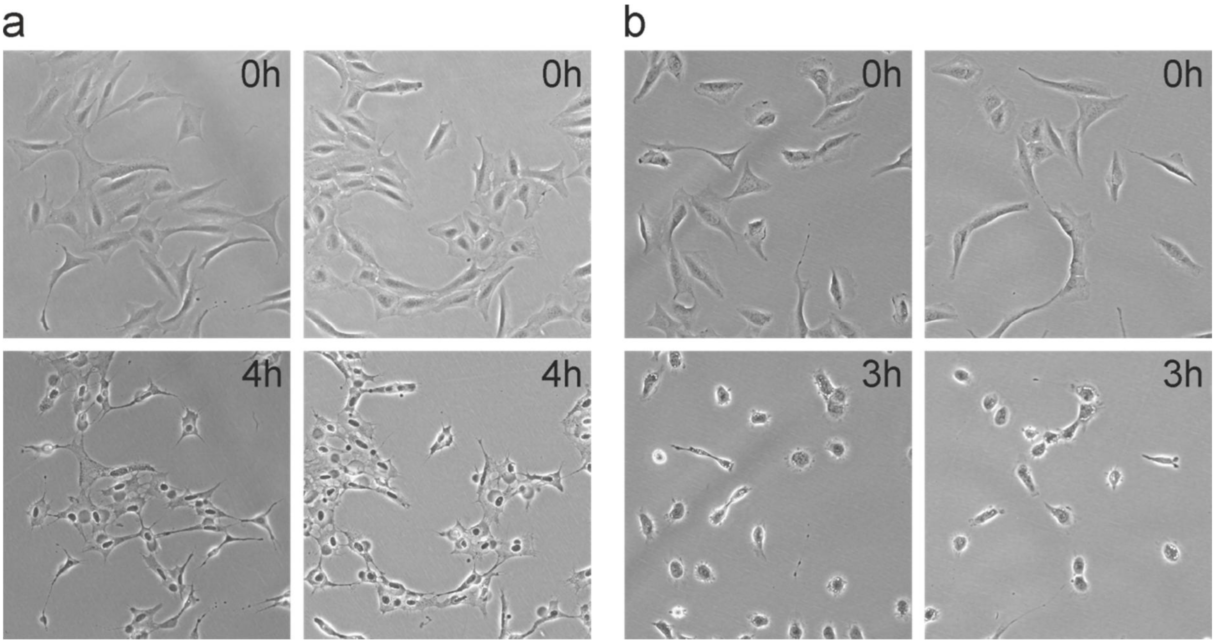

Supplementary Figure 6. Incubation of U2OS cells with switching buffer components. (**a**) Cells were incubated with DMEM Ham's F12 supplemented with 15 mM HEPES and oxygen scavenger (4% glucose, 8 U/ml glucose oxidase, 160 U/ml catalase) for 20 min at RT. Afterwards the buffer was replaced with DMEM Ham's F12 complete growth medium and incubated at 37°C and 5% CO_2. Upper panels show cells immediately after buffer incubation (0 h) and lower panels show the same cells after 4 h of observation. (**b**) Cells were incubated with DMEM Ham's F12 supplemented with 15 mM HEPES and 100 mM glutathione at 37°C and 5% CO_2. Upper panels indicate the begin of the incubation (0 h) and lower panels show the same cells after 3 h. Stressed cells show shrinking and detachment. Depending on the concentration, thiols can scavenge oxygen as well[1]. With 50 mM glutathione, cells did not show obvious morphological changes (data not shown).

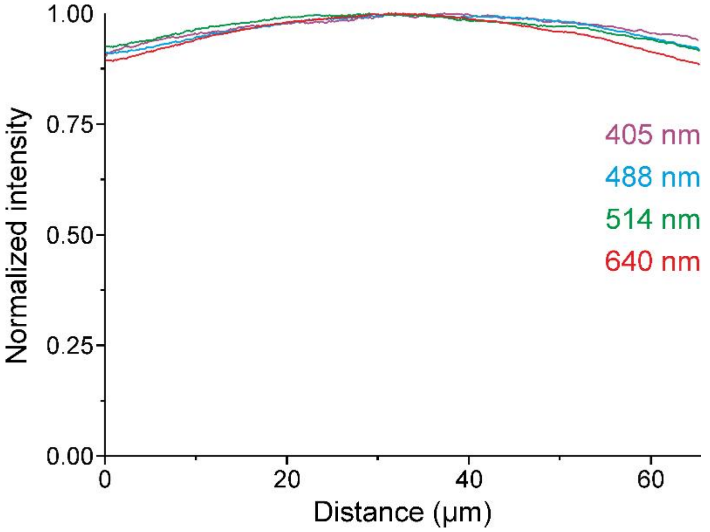

Supplementary Figure 7. Laser intensity profiles. The profile was measured by irradiating a 10^{-6} M dye solution followed by fluorescence detection. The laser beam was confined with a rectangular field stop defining an illuminated field of view of 65.5 µm x 65.5 µm. Laser beams were largely expanded to achieve a marginal intensity drop of 6-12% from the maximum value in the center to the edge.

Supplementary Videos 1-3. Classification of photodamage effects using U2OS cells in three categories. (1) Non-irradiated healthy cells (**Figure 1a**), (2) apoptotic cells irradiated with an intensity of 0.49 kW cm^{-2} at 514 nm for 240 s (**Figure 1b**), and (3) frozen cells irradiated with an intensity of 1.5 kW cm^{-2} at 514 nm for 240 s (**Figure 1c**). Videos were recorded after irradiation in an automated cell observation system. The red rectangle at the beginning shows the irradiated cells. Scale bar, 50 µm.

REFERENCES

1. Schafer, P., van de Linde, S., Lehmann, J., Sauer, M. & Doose, S. Methylene blue- and thiol-based oxygen depletion for super-resolution imaging. *Anal. Chem.* **85**, 3393-3400 (2013).
